# Application of a derivative of human defensin 5 to treat ionizing radiation-induced enterogenic infection

**DOI:** 10.1093/jrr/rrad104

**Published:** 2024-01-23

**Authors:** Gaomei Zhao, Yingjuan He, Yin Chen, Yiyi Jiang, Chenwenya Li, Tainong Xiong, Songling Han, Yongwu He, Jining Gao, Yongping Su, Junping Wang, Cheng Wang

**Affiliations:** State Key Laboratory of Trauma and Chemical Poisoning, Chongqing Engineering Research Center for Nanomedicine, College of Preventive Medicine, Institute of Combined Injury of PLA, Third Military Medical University, Gaotanyan Street No. 30, Shapingba District, Chongqing 400038, China; State Key Laboratory of Trauma and Chemical Poisoning, Chongqing Engineering Research Center for Nanomedicine, College of Preventive Medicine, Institute of Combined Injury of PLA, Third Military Medical University, Gaotanyan Street No. 30, Shapingba District, Chongqing 400038, China; State Key Laboratory of Trauma and Chemical Poisoning, Chongqing Engineering Research Center for Nanomedicine, College of Preventive Medicine, Institute of Combined Injury of PLA, Third Military Medical University, Gaotanyan Street No. 30, Shapingba District, Chongqing 400038, China; State Key Laboratory of Trauma and Chemical Poisoning, Chongqing Engineering Research Center for Nanomedicine, College of Preventive Medicine, Institute of Combined Injury of PLA, Third Military Medical University, Gaotanyan Street No. 30, Shapingba District, Chongqing 400038, China; State Key Laboratory of Trauma and Chemical Poisoning, Chongqing Engineering Research Center for Nanomedicine, College of Preventive Medicine, Institute of Combined Injury of PLA, Third Military Medical University, Gaotanyan Street No. 30, Shapingba District, Chongqing 400038, China; State Key Laboratory of Trauma and Chemical Poisoning, Chongqing Engineering Research Center for Nanomedicine, College of Preventive Medicine, Institute of Combined Injury of PLA, Third Military Medical University, Gaotanyan Street No. 30, Shapingba District, Chongqing 400038, China; State Key Laboratory of Trauma and Chemical Poisoning, Chongqing Engineering Research Center for Nanomedicine, College of Preventive Medicine, Institute of Combined Injury of PLA, Third Military Medical University, Gaotanyan Street No. 30, Shapingba District, Chongqing 400038, China; State Key Laboratory of Trauma and Chemical Poisoning, Chongqing Engineering Research Center for Nanomedicine, College of Preventive Medicine, Institute of Combined Injury of PLA, Third Military Medical University, Gaotanyan Street No. 30, Shapingba District, Chongqing 400038, China; State Key Laboratory of Trauma and Chemical Poisoning, Chongqing Engineering Research Center for Nanomedicine, College of Preventive Medicine, Institute of Combined Injury of PLA, Third Military Medical University, Gaotanyan Street No. 30, Shapingba District, Chongqing 400038, China; State Key Laboratory of Trauma and Chemical Poisoning, Chongqing Engineering Research Center for Nanomedicine, College of Preventive Medicine, Institute of Combined Injury of PLA, Third Military Medical University, Gaotanyan Street No. 30, Shapingba District, Chongqing 400038, China; State Key Laboratory of Trauma and Chemical Poisoning, Chongqing Engineering Research Center for Nanomedicine, College of Preventive Medicine, Institute of Combined Injury of PLA, Third Military Medical University, Gaotanyan Street No. 30, Shapingba District, Chongqing 400038, China; State Key Laboratory of Trauma and Chemical Poisoning, Chongqing Engineering Research Center for Nanomedicine, College of Preventive Medicine, Institute of Combined Injury of PLA, Third Military Medical University, Gaotanyan Street No. 30, Shapingba District, Chongqing 400038, China

**Keywords:** ionizing radiation, enterogenic infection, human defensin, intestinal injury, gut microbiota

## Abstract

Enterogenic infection is a common complication for patients with radiation injury and requires efficient therapeutics in the clinic. Herein, we evaluated the promising drug candidate ^T7E21R^HD5, which is a peptide derived from intestinal Paneth cell-secreted human defensin 5. Oral administration of this peptide alleviated the diarrhea symptoms of mice that received total abdominal irradiation (TAI, γ-ray, 12 Gy) and improved survival. Pathologic analysis revealed that ^T7E21R^HD5 elicited an obvious mitigation of ionizing radiation (IR)-induced epithelial damage and ameliorated the reduction in the levels of claudin, zonula occluden 1 and occludin, three tight junction proteins in the ileum. Additionally, ^T7E21R^HD5 regulated the gut microbiota in TAI mice by remodeling β diversity, manifested as a reversal of the inverted proportion of *Bacteroidota* to *Firmicutes* caused by IR. ^T7E21R^HD5 treatment also decreased the abundance of pathogenic *Escherichia–Shigella* but significantly increased the levels of *Alloprevotella* and *Prevotellaceae_NK3B31*, two short-chain fatty acid-producing bacterial genera in the gut. Accordingly, the translocation of enterobacteria and lipopolysaccharide to the blood, as well as the infectious inflammatory responses in the intestine after TAI, was all suppressed by ^T7E21R^HD5 administration. Hence, this versatile antimicrobial peptide possesses promising application prospects in the treatment of IR-induced enterogenic infection.

## INTRODUCTION

The widespread application of nuclear energy and technology increases the risk of radiation injuries. If given a high dose of ionizing radiation (IR), people may suffer from acute radiation sickness (ARS) that presents as multisystemic symptoms including hematopoietic dysfunction, bleeding, infection and metabolic disorders, in the clinic [1]. Infection is a common complication of ARS and threatens to the lives of patients with radiation injury [2]. ARS-related infection is due to exogenous and endogenous sources and demonstrates shortened latency and transformation of conditional pathogenic microbes to pathogens [3]. Because the gut is one of the most sensitive organs to irradiation and contains a large number of conditional pathogenic microorganisms, IR-induced endogenous infection often originates from the intestine [4]. Coping with enterogenic infection is thus an important measure for ARS treatment, but effective drugs are currently extremely deficient.

In addition to mucosal barrier damage, gut microbiota dysbiosis is a primary cause of IR-induced enterogenic infection. It has been reported that abdominal radiation elicits gut dysbiosis and decreases the diversity of microbiota in mice [5]. The abundances of aerobic bacteria and *Lactobacillus* in the intestine significantly decrease 2 h after IR [6]. Similarly, decreased α diversity and increased β diversity are observed in radiation enteritis patients [7]. Previous study has demonstrated that IR-induced microbiota dysbiosis correlated with the occurrence of symptoms, including diarrhea, fatigue and systemic inflammatory response, in cancer patients who received pelvic radiotherapy [8]. Generally, the trend of microbiota change manifests as the inverted proportion of *Bacteroidota* to *Firmicutes* and an increased abundance of pathogenic bacteria [4]. Such pathological alterations provide for the translocation of conditional pathogens and correlative toxins such as Gram-negative bacteria-derived lipopolysaccharides (LPS), resulting in the pathogenesis of endotoxemia, systemic inflammation and multiple organ failure [9].

Antimicrobial peptides (AMPs) are important components of natural immunity in animals and plants. To address the microbial challenges in the gut, the human intestine has evolved to produce a range of AMPs including human defensin 5 (HD5) [10], a small and Cys-rich peptide specifically secreted by Paneth cells [11]. Because IR destroys Paneth cells in a dose-dependent manner [12], HD5 reduction may contribute to microbiota dysbiosis. Conversely, exogenous peptide supplementation is a potential measure for maintaining gut homeostasis after IR. Nevertheless, due to the ‘shielding effect’ donated by high concentrations of salt ions and anionic proteins, the suppressive effect of exogenous AMPs on microbes is weakened *in vivo* [13, 14]. To promote the application prospect of HD5, site mutations around its C-terminal active region using an adaptive evolutionary strategy were performed, obtaining a derivative termed ^T7E21R^HD5 that was resistant to the ‘shielding effect’ and protease degradation in the intestine [15].

Herein, we evaluated the impact of oral administration of ^T7E21R^HD5 on IR-induced enterogenic infection. A total abdominal irradiation (TAI) mouse experiment was performed. Pathologic analysis was carried out to observe the intestinal mucosal destruction. The incidence of bacteremia and LPS content in the serum were determined. Furthermore, mouse feces were collected for 16S rDNA sequencing to analyze the role of the peptide in microbiota regulation. Our study demonstrates that ^T7E21R^HD5 can mitigate IR-induced enterogenic infection and thus provides an instrumental drug candidate for ARS treatment.

## MATERIALS AND METHODS

### Peptide preparation

^T7E21R^HD5 was fabricated by the Chinese Peptide Company (Hangzhou, Zhejiang Province, China) using solid-phase chemical synthesis. The detailed amino acid sequence, purity and molecular mass of the prepared product are presented in Fig. S1.

### TAI mouse experiment

**Fig. 1:**
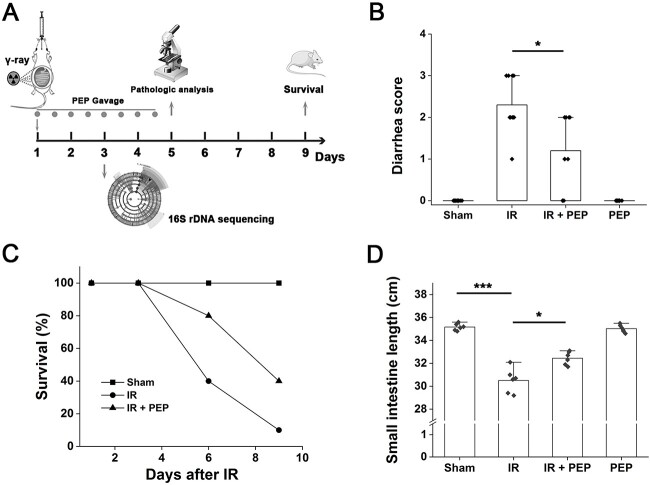
Effect of ^T7E21R^HD5 treatment on the survival of mice after TAI. (A) Schematic diagram illustrating the experiment design. (B) Diarrhea scores of mice on Day 5 after TAI. Results are shown as means ± standard deviation (SD). ^*^P < 0.05. (C) Mice survival on Days 1, 3, 6 and 9 after TAI. Each group contains 10 mice. (D) Small intestine length of TAI mice in the absence and presence of peptide treatment. ^*^P < 0.05; ^***^P < 0.001.

Male 8-week-old C57 mice weighing 20–22 g were obtained from the Experimental Animal Center of our institution and randomly divided into four groups (*n* = 10): (i) sham group, (ii) IR + PBS, (iii) IR + PEP (^T7E21R^HD5) and (iv) PEP group. The mice were cared for and treated in accordance with the recommendations in the National Institutes of Health (NIH) Guide for the Care and Use of Laboratory Animals (NIH Publication No. 85e23 Rev. 1985). Mice placed in the supine position after anesthesia were irradiated with a ^60^Co source of 12 Gy γ-ray at a dose rate of 0.45 Gy/min. Sterile PBS (Group 2) or ^T7E21R^HD5 (30 μg, Group 3) was given after IR by gavage twice a day for 4 days (Fig. 1A). Nonirradiated mice in Groups 1 and 4 were orally administered PBS and ^T7E21R^HD5, respectively. Diarrhea symptom scores and body weight were determined on Day 5 after TAI as previously described [16].

For pathologic analysis, another 24 mice (*n* = 6) were used to repeat the TAI experiment. Mice were sacrificed on Day 5 after TAI, and their intestinal tissues and blood samples were obtained by surgery. The lengths of the mouse small intestine and colon were measured. Intestinal morphology was observed after hematoxylin & eosin (HE) staining (C0105S; Beyotime, Shanghai, China), by which the ileal villous height and the number of crypts were determined. Mouse intestinal goblet cells were visualized by periodic acid–Schiff (PAS) staining (C0142S, Beyotime). Tight junction proteins, including occludin, claudin and zonula occluden 1 (ZO-1) in the intestinal epithelium, were examined using immunofluorescence staining. The TAI mouse experiment was approved by the Animal Experimental Ethics Committee of our institution.

### Immunofluorescence staining

Paraffin sections of mouse ileum tissues were deparaffinized using dimethylbenzene and ethyl alcohol, followed by antigen retrieval in boiling ethylene diamine tetraacetic acid (EDTA) solution (pH 8.0). Nonspecific epitopes were blocked in 1% bovine serum albumin (BSA) for 30 min. The sections were incubated with a rabbit polyclonal anti-occludin antibody (AF300990, Aifang Biological, Changsha, Hunan Province, China, 1:1000), a rabbit polyclonal anti-claudin-1 antibody (AF20044, Aifang Biological, 1:1000) and a rabbit polyclonal anti-ZO-1 antibody (AF09017, Aifang Biological, 1:1000). Goat anti-rabbit Alexa Fluor 488 (A0423, Beyotime) or donkey anti-rabbit Alexa Fluor 525 (A0453, Beyotime) was applied to visualize the locations of these tight junction proteins in the intestine. Images were obtained on a Zeiss LSM 780 NLO confocal microscope. Quantitative analysis was conducted using ImageJ.

### Limulus amebocyte lysate assay

Mouse blood obtained on Day 5 post TAI was processed by centrifugation at 3000 rpm  for 15 min to collect the serum. A total of 100 μl of mouse serum was coincubated with 100 μl of tachypleus amebocyte lysate (EC80545, Xiamen Bioendo Technology, Fujian Province, China) at 37°C for 10 min, followed by the addition of 100 μl of chromogenic matrix solution. After coincubation at 37°C for 6 min, hydrochloric acid solution was added to stop the reaction. Azo reagent was finally pipetted into the mixture, the absorbance of which at 545 nm was then determined using a Molecular Devices M2e microplate reader (Sunnyvale, CA). This experiment was conducted in triplicate and repeated three times.

### Bacteremia detection

One hundred microliters of mouse blood collected on Day 5 after TAI were coincubated with 10 ml of sterile Mueller-Hinton broth in an aerobic environment. Additionally, a total of 400 μl of mouse blood was added to an anaerobic bacteria culture bottle (bioMérieux, Marcy l’Etoile, France). The mixture was then cultured in a constant temperature shaker (200 rpm) at 37°C for 24 h. Turbidity of nutrient medium was considered bacteremia.

### Quantitative polymerase chain reaction

RNA from the mouse ileum was extracted with TaKaRa (Dalian, Shandong Province, China) RNAiso Plus reagent and reverse transcribed using a TaKaRa PrimeScript™ RT–PCR kit (DRR014A). Forward (F) and reverse (R) primers were applied in the following polymerase chain reactions (PCRs): IL-1β, 5′-TCTCGCAGCAGCACATCA-3′ (F), 5′-CACACACCAGCAGGTTAT-3′ (R); IL-6, 5′-TGGGAAATCGTGGAAATGAG-3′ (F), 5′-CTCTGAAGGACTCTGGCTTTG-3′ (R) and GAPDH, 5′-CGGTGCTGAGTATGTCGTGGAGTC-3′ (F), 5′-GGGGCTAAGCAGTTGGTGGTG-3′ (R). Data were recorded on a Bio-Rad iQ5 standard edition optical system software (version 2.1). This experiment was conducted in triplicate and repeated three times.

### 16S rDNA sequencing

On Day 3 post irradiation, the fecal samples of six mice for each group were collected for 16S rDNA sequencing. Genomic DNA was extracted using a Magnetic Soil and Stool DNA Kit (DP712, TianGen, Beijing, China). The 16S rRNA genes of the V3–V4 regions were amplified with Phusion High-Fidelity PCR Master Mix (M0531L, New England Biolabs, Beijing, China). The TianGen Universal DNA Purification Kit (DP214) was used to purify the PCR products. Library preparation, sequencing, amplicon sequence variant (ASV) denoising and species annotation were carried out by Novogene (Beijing, China). Alpha diversity indices, including Chao1, observed features, Shannon and Simpson indices, were calculated using QIIME2 software. Nonmetric multidimensional scaling (NMDS) was conducted using R software (Version 4.0.3) with the ade4 package and ggplot2 package. To reveal the community structure differentiation, a *t*-test was employed to identify species with significant differences between groups at various classification levels.

### Statistical analysis

Statistical analysis was performed by SPSS Statistics 17.0 using the LSD multiple-comparison test and Student’s *t*-test. *P* < 0.05 was considered to be statistically significant.

## RESULTS

### ^T7E21R^HD5 treatment improves the survival of irradiated mice

To evaluate the influence of ^T7E21R^HD5 treatment on IR-induced enterogenic infection, we established a mouse model induced by TAI using a 12 Gy γ-ray. ^T7E21R^HD5 was orally administered twice a day post irradiation (Fig. 1A). After continuous drug administration for 4 days, diarrhea symptoms were scored. Obvious diarrhea was observed in irradiated mice, but the symptom was significantly mitigated by ^T7E21R^HD5 (Fig. 1B, *P* < 0.05). Consistently, mouse body weight was also recorded, and a significant difference was determined between IR and IR + PEP groups (*P* < 0.05, Fig. S2). Approximately 90% of the irradiated mice (*n* = 10) died after 9 days, whereas the survival increased to 40% in ^T7E21R^HD5-treated mice (Fig. 1C). We next repeated the TAI experiment using another 24 mice (*n* = 6). On Day 5 post TAI, mice were sacrificed and the intestinal tissues were obtained. Measurement of the small intestine length showed that due to the intestinal shrinkage caused by IR damage, TAI markedly decreased the tissue length compared with that in the sham group (Fig. 1D, *P* < 0.001). Nevertheless, ^T7E21R^HD5 enabled a reversal of the pathological change (*P* < 0.05). Similar results were determined in terms of the colon length (Fig. S3).

### ^T7E21R^HD5 mitigates intestinal mucosal destruction caused by IR

**Fig. 2:**
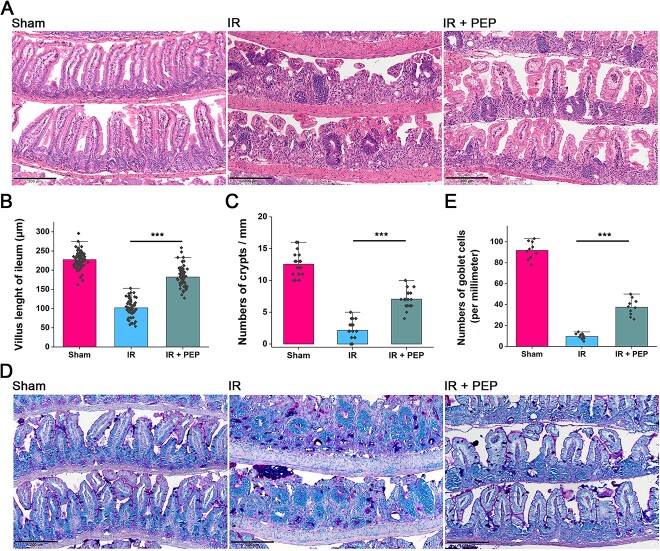
Histopathological analysis on mouse intestinal epithelium after TAI. (A) HE staining revealing the mitigation of ^T7E21R^HD5 on the intestinal mucosal destruction caused by IR. Scale indicates 200 μm. (B) Counts of mouse ileal villous height. Results are shown as means ± SD. ^***^P < 0.001. (C) Number of crypts in mouse ileum in the presence and absence of TAI and peptide treatment. Results are shown as means ± SD. ^***^P < 0.001. (D) PAS staining showing goblet cells in mouse ileum. Scale indicates 200 μm. (E) Counts of goblet cells in mouse ileum after TAI and peptide treatment. Results are shown as means ± SD. ^***^P < 0.001.

To probe the alleviative effect of ^T7E21R^HD5, we further carried out histopathological analysis on the obtained intestinal tissues. HE staining revealed a severe acute intestinal injury in irradiated mice, manifested by apparent villous shedding (Fig. 2A). Encouragingly, oral administration of ^T7E21R^HD5 elicited an obvious mitigation of IR-triggered epithelial damage, as indicated by the maintenance of ileal villous height after TAI (Fig. 2B, *P* < 0.001). Additionally, more crypts were counted in the ileum of mice treated with ^T7E21R^HD5 than in TAI mice (Fig. 2C, *P* < 0.001). Since goblet cells produce mucins and regulate epithelial renewal [17], we also carried out PAS staining and discovered that the number of goblet cells was notably reduced in irradiated mice (Fig. 2D), whereas ^T7E21R^HD5 administration dramatically increased the number of goblet cells in the ileum (Fig. 2E, *P* < 0.001).

**Fig. 3:**
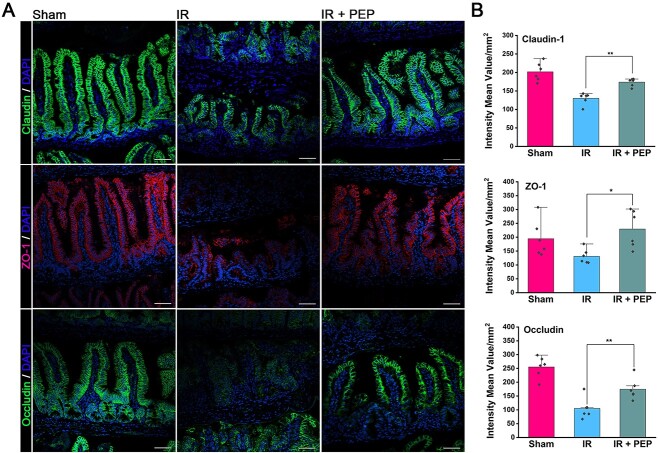
Amelioration of ^T7E21R^HD5 on the reduction of intestinal tight junction proteins induced by IR. (A) Confocal images indicating the expressions of claudin, ZO-1 and occludin in mouse ileum. Nucleus is stained by 4,6-diamino-2-phenyl indole (DAPI). The scale bar indicates 20 μm. (B) Mean fluorescence intensity of the three intestinal tight junction proteins. Results are presented as the means ± SD. ^*^P < 0.05; ^**^P < 0.01.

Furthermore, immunofluorescence was conducted to observe the expressions of claudin, ZO-1 and occludin, three tight junction proteins contributing to the formation of the intestinal epithelial barrier [18]. IR markedly decreased the levels of these proteins in the ileum (Fig. 3A) and colon (Fig. S4). Similarly, the reduction in these proteins was visibly ameliorated through ^T7E21R^HD5 administration (Fig. 3B). These findings suggest that ^T7E21R^HD5 treatment mitigates intestinal mucosal destruction induced by IR.

### ^T7E21R^HD5 decreases IR-induced intestinal infection

**Fig. 4:**
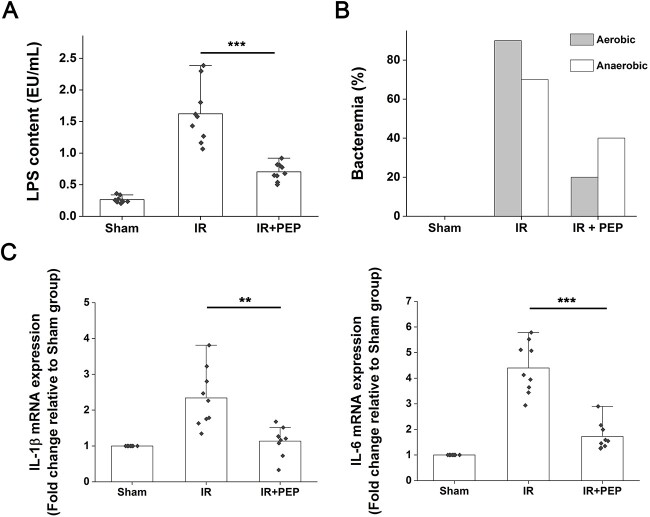
Mitigative effect of ^T7E21R^HD5 treatment on the enterogenic infection caused by IR. (A) Determination of LPS levels in mouse serum. Results are presented as the means ± SD. ^***^P < 0.001. (B) Incidence of bacteremia in TAI mice in the presence and absence of peptide treatment. (C) Alleviation of ^T7E21R^HD5 on IR-induced upregulations of IL-1β and IL-6 in mouse ileum. Results are shown as the means ± SD. ^**^P < 0.01; ^***^P < 0.001.

Intestinal barrier dysfunction allows the translocation of intrinsic toxins and microbes [19]. Because ^T7E21R^HD5 could inhibit the destruction of the gut mucosa, we next speculated an alleviation of the peptide against endogenous intestinal infection. Given a healthy barrier function, bacterial LPS cannot infiltrate into the blood. Our limulus amebocyte lysate assay showed that IR elicited a high LPS level in mouse serum on Day 5 post TAI, whereas ^T7E21R^HD5 administration significantly decreased LPS translocation (Fig. 4A, *P* < 0.001). Consistently, the occurrence of bacteremia originating from enterogenous aerobic and anaerobic bacteria after IR was decreased by ^T7E21R^HD5 (Fig. 4B). Determination of the mRNA expression levels of proinflammatory cytokines, including IL-1β and IL-6, in the ileum also supported that ^T7E21R^HD5 treatment was instrumental in reducing the infective inflammatory responses (Fig. 4C).

### ^T7E21R^HD5 regulates gut microbiota in irradiated mice

**Fig. 5:**
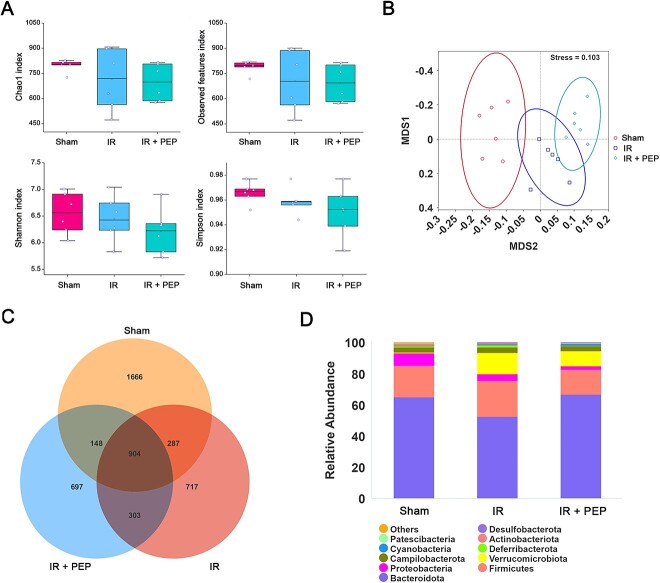
Influence of TAI and ^T7E21R^HD5 treatment on the diversity of mouse microbiota. (A) Chao1, observed features, Shannon and Simpson indices illustrating α diversity of mouse microbiota. (B) NMDS showing β diversity of mouse microbiota. (C) Venn diagram indicating the abundance of ASV in each group. (D) Species abundance histogram showing the top 10 bacterial species at the phylum level.

In addition to the protective effect on the intestinal epithelial barrier, we also evaluated the role of ^T7E21R^HD5 in regulating the gut microbiota. Bacterial DNA with high purity (OD_260_:OD_280_ = 1.8–2.0) was extracted from mouse feces. The 16S rDNA sequencing obtained 4722 ASVs from 18 samples. The α diversity indices, including Chao1, observed features, Shannon and Simpson indices, were analyzed, but no significances were determined (Fig. 5A), suggesting that the abundance and evenness of the gut microbiota were not significantly influenced by IR and ^T7E21R^HD5 treatment after 2 days. Nevertheless, the nonparametric test Anosim analysis revealed that the level of intergroup difference was markedly greater than that within the group, as manifested by a *P* value lower than 0.05 (*P* = 0.01, R = 0.159). NMDS was also performed and found a separation of the gut microbiota before and after exposure to IR or ^T7E21R^HD5 with a stress value of 0.103 (Fig. 5B), indicative of the influence of irradiation and ^T7E21R^HD5 on β diversity of the mouse microbiota.

**Fig. 6:**
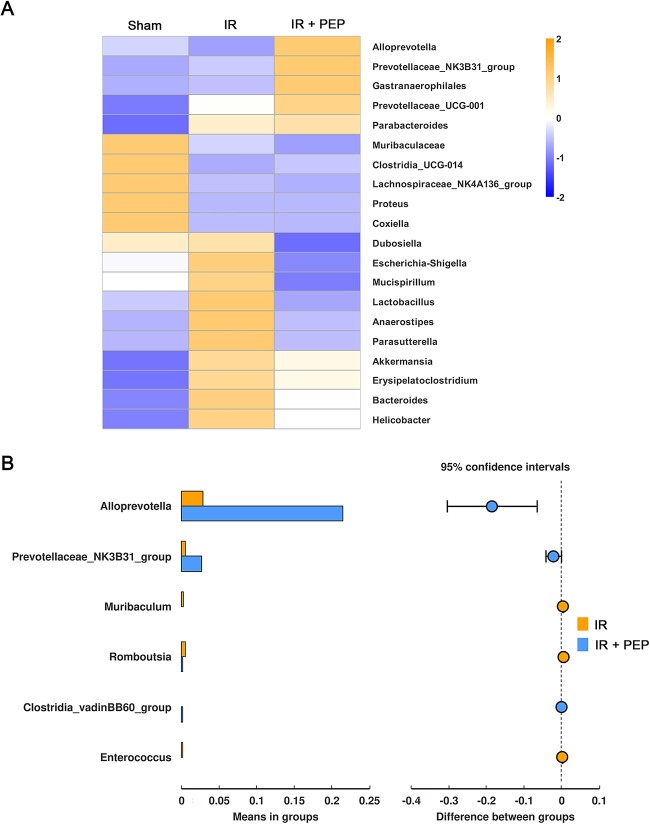
Regulation of ^T7E21R^HD5 on the species abundance of mouse microbiota. (A) Clustering heat map showing the alteration of top 20 species after TAI and peptide treatment. (B) *t*-test analysis on the role of ^T7E21R^HD5 in regulating mouse microbiota after TAI. Shown are the species significantly changed in TAI mice after peptide treatment.

Despite the shared 904 ASVs, the number of specific ASVs in the sham group, IR group and ^T7E21R^HD5 treatment group was 1666, 717 and 697, respectively (Fig. 5C). Based on the species annotation results, we initially identified the top 10 bacterial species at the phylum level. *Bacteroidota* and *Firmicutes* were the two phyla with the highest relative abundance in healthy mice (Fig. 5D). Irradiation decreased the abundance of *Bacteroidota* from 64.96 to 52.31% but increased the *Firmicutes* content from 20.24 to 23.19% (Table S1). Additionally, the level of *Verrucomicrobiota* was increased from 0.78 to 13.46% post TAI. Oral administration of ^T7E21R^HD5 elicited an abundance of 66.62% for *Bacteroidota*, 16.1% for *Firmicutes* and 9.59% for *Verrucomicrobiota*, suggesting that ^T7E21R^HD5 could reverse the gut microbiota disorder caused by IR. Further analysis at the genus level showed that *Muribaculaceae*, *Alloprevotella*, *Lactobacillus*, *Coxiella* and *Dubosiella* were the five dominant strains in healthy mice (Fig. S5). Nevertheless, homeostasis was disrupted by IR, with a reduced abundance of *Muribaculaceae* but an increased level of *Escherichia–Shigella* (Fig. 6A). Encouragingly, ^T7E21R^HD5 treatment not only decreased the levels of *Escherichia–Shigella* but also significantly increased the levels of *Alloprevotella* (*P* = 0.01, Fig. 6B) and *Prevotellaceae_NK3B31* (*P* = 0.041) in TAI mice.

## DISCUSSION

IR-induced enterogenic infection originates from the translocation of intestinal microorganisms and their produced toxins to the circulation, resulting in systemic infection or endotoxemia that seriously threatens to human life and health [20]. Intestinal epithelial cells, the mucous layer and symbiotic microorganisms are the primary components of the gut barrier. A large dose of IR can directly destroy cellular nucleic acids and initiate excess activation of programmed cell death pathways including apoptosis, necrosis, pyroptosis and ferroptosis [21, 22], causing epithelial necrosis and detachment, which allows pathogens to enter the body. In the clinic, IR-induced enterogenic infection primarily occurs in abdominal or pelvic tumor patients after radiotherapy, where extensive shedding of the intestinal epithelium rarely occurs. However, microbiota dysbiosis and the loss of epithelial connections caused by a reduction in tight junction proteins are contributable [8]. Despite the efficacy in preventing microbial invasion, accumulating evidence suggests that traditional antibiotic administration has negative effects on gut microbiota composition [23–25]. Alternatively, we provided a mild candidate to treat IR-induced enterogenic infection in this study.

Intestinal defensins, a group of AMPs secreted by enterocytes and Paneth cells, are distributed in the mucus layer and enteric cavity and constitute a crucial line of defense against microbial invasion [26]. Human Paneth cells produce two evolutionally conserved defensin peptides, HD5 and HD6 [11, 27]. In contrast to the weak bacterial killing property of HD6, HD5 exhibits antimicrobial actions by binding to anionic targets on the bacterial surface, such as lipid A and lipoteichoic acid [26], and penetrating the outer and inner membranes consecutively [28]. ^T7E21R^HD5 is derived from HD5 by replacing adaptive evolution sites Thr^7^ and Glu^21^ with Arg, an electropositive amino acid highly selected in human α defensins [29]. Because ^T7E21R^HD5 presents as an atypical dimer with parallel β strands, which elicits a larger cationic clawlike active region than HD5, ^T7E21R^HD5 is more efficient than HD5 in membrane recruitment and bacterial inactivation, particularly in saline and serum solutions. Thus, it possesses better prospects for drug development [15]. Delivery of ^T7E21R^HD5 to the intestine has been previously confirmed to effectively eliminate exogenous pathogenic drug-resistant bacteria and mitigate intestinal inflammation by reducing the proinflammatory factors TNF-α, IL-1β and MMP-9 *in vivo* [30]. Our data showed that ^T7E21R^HD5 administration was also able to alleviate endogenous infection and inflammatory responses caused by IR, thus demonstrating a versatile peptide in infection treatment.

Tight junction proteins including occludin, claudin and ZO-1, which join adjacent cell membranes to form a solid physical barrier, are crucial components of the intestinal defense against pathological organisms [31]. Due to its role in maintaining cell polarity and epithelial integrity, disruption of tight junctions is considered an early hallmark of intestinal epithelial injury. Luminal toxins such as LPS, which is derived from the outer membrane of Gram-negative bacteria, negatively regulate the expression of tight junction proteins, possibly through an inflammatory signaling cascade [32]. The Akt signaling pathway is also involved in junction compromise caused by LPS [33, 34]. Radiotherapy changed the ratio of *Bacteroidota* to *Firmicutes* and increased LPS content in the enteric cavity [8]. Our findings supported that IR elicited an imbalance of intestinal flora with an increased abundance of *Escherichia–Shigella*, a Gram-negative bacterium that can release LPS and is associated with enterogenous sepsis [35]. Because oral administration of ^T7E21R^HD5 efficiently eliminates pathogenic *Escherichia in vivo* [30], it is plausible that ^T7E21R^HD5 treatment can reduce the level of *Escherichia–Shigella* and increase occludin, claudin and ZO-1 expression levels in irradiated mice.

The composition of the gut microbiota in healthy people is stable and in a dynamic equilibrium state. An increasing number of studies have suggested that the imbalance of intestinal flora is a key cause of enterogenic infection. Comparison of susceptibility to IR between germ-free mice and wild-type mice directly reflects the importance of the gut microbiota, raising the possibility of treating IR-induced enterogenic infection through microbiota regulation [36, 37]. In line with previous findings that a single dose of γ-ray irradiation did not influence α diversity of mouse intestinal flora after 3 days [38], no significant difference was observed between healthy and irradiated mice in our study. Xiao *et al.* also found that the observed species number of mouse enteric bacteria was not significantly changed at Days 6 and 12 after a 12 Gy of TAI [37]. However, a decreased α diversity was discovered in TAI-treated mice after 3 month [5]. It seemed that the α diversity was an indicator altered at the late stage of IR-induced intestinal injury. Comparatively, the β diversity of mouse gut microbiota changed early after TAI. We detected a reduction in the abundance of *Bacteroidota* and an increase in the proportions of *Firmicutes* and *Verrucomicrobiota* on Day 3 post irradiation. Similar transformation was also discovered in mice received a total body 9.5 Gy γ-irradiation after 24 h [9].

*Bacteroides* and *Firmicutes* are the most abundant species in human gut microbiota. *Firmicutes*/*Bacteroidetes* (F/B) ratio is an important index reflecting the composition of gut microbiota and undergoes changes with alterations in drug administration, dietary habit, physical activity and pathological conditions [39]. An reduced F/B ratio was found in patients with inflammatory bowel diseases [40] and Type 1 diabetes [41], while an increased ratio correlated to the pathogenesis of obesity [42] and IR-induced intestinal injury [4]. To modulate the F/B ratio to an appropriate range, some strategies including probiotics, prebiotics and fecal transplantation have been developed [39]. In the present study, we provided a peptide regulator ^T7E21R^HD5, which not only corrected the abnormal F/B ratio caused by IR but also enhanced the colonization of *Alloprevotella* and *Prevotellaceae*, two short-chain fatty acid (SCFA)-producing bacterial genera [43]. The increase in *Alloprevotella* and *Prevotellaceae* has been reported to enhance the contents of propionate and butyrate in the gut [44]. As two widely known SCFAs, the protective effect of propionate and butyrate on intestinal injury is well studied. Propionate attenuates intestinal inflammation by inhibiting NF-κB activation *via* G-protein coupled receptor 41 (GPR41) [45]. Mice given propionate are resistant to intestinal injury induced by a lethal dose of IR [46]. Moreover, butyrate ameliorates enterogenous sepsis by suppressing histone deacetylases and decreasing NF-κB p65 nuclear translocation [47]. Accordingly, enhancement of probiotics by ^T7E21R^HD5 also benefits the alleviation of IR-induced enterogenic infection.

## CONCLUSION

We demonstrate the application prospect of ^T7E21R^HD5, a rationally designed molecule based on human intestinal defensin, in irradiation injury therapy. ^T7E21R^HD5 administration mitigates intestinal mucosal destruction caused by IR and decreases the translocation of endogenous bacteria and LPS, which alleviates local inflammatory responses and improves the survival of irradiated mice. Additionally, ^T7E21R^HD5 can regulate the microbiota dysbiosis post IR by enhancing the abundance of probiotics that are instrumental for suppressing enterogenic infection. Considering the shortage of efficient therapeutics against IR-induced enterogenic infection, the versatile AMP is a promising drug candidate.

## CONFLICT OF INTEREST

The authors declare that they have no conflicts of interest.

## FUNDING

This study was supported by grant from the National Natural Science Foundation of China (Nos. 82003398 and 82003395).

## Supplementary Material

Supplementary_Materials_rrad104
