# Supplementary material for: Application of a derivative of human defensin 5 to treat ionizing radiation-induced enterogenic infection
Source: J Radiat Res. 2024 Jan 23;65(2):194–204. doi: 10.1093/jrr/rrad104 (PMC10959430; doi:10.1093/jrr/rrad104)
Supplement: Supplementary_Materials_rrad104 [file supplementary_materials_rrad104.docx]

# Supplementary Materials

**Application of a derivative of human defensin 5 to treat** **ionizing radiation-induced** **enterogenic infection**

**Contents:**

**Table S1.** Relative abundance of top 5 bacterial species at the phylum level

**Figure S1.** Amino acid sequence, purity, and molecular mass of ^T7E21R^HD5

**Figure S2.** Body weights of mice on Day 5 after TAI

**Figure S3.** Colon length of TAI mice in the absence and presence of ^T7E21R^HD5 treatment

**Figure S4.** Amelioration of ^T7E21R^HD5 on the reduction of tight junction proteins in colon induced by IR

**Figure S5.** Species abundance histogram showing the top 10 bacterial species at the genus level

**Table S1.** Relative abundance of top 5 bacterial species at the phylum level

| Taxonomy | Bacteroidota | Firmicutes | Proteobacteria | Verrucomicrobiota | Campilobacterota |
| --- | --- | --- | --- | --- | --- |
| Sham | 64.96% | 20.24% | 7.89% | 0.78% | 2.9% |
| IR | 52.31% | 23.19% | 4.41% | 13.46% | 3.53% |
| IR+PEP | 66.62% | 16.1% | 2.18% | 9.59% | 3.22% |


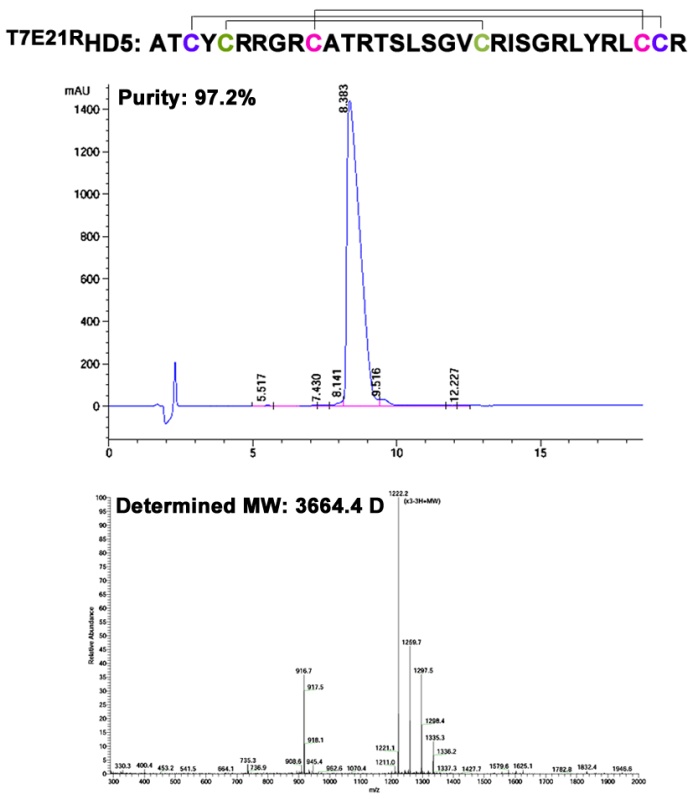


**Figure S1.** Amino acid sequence, purity, and molecular mass of ^T7E21R^HD5. The purity of the peptide was confirmed over 95%. The determined molecular weight of ^T7E21R^HD5 matched closely with its theoretical value.


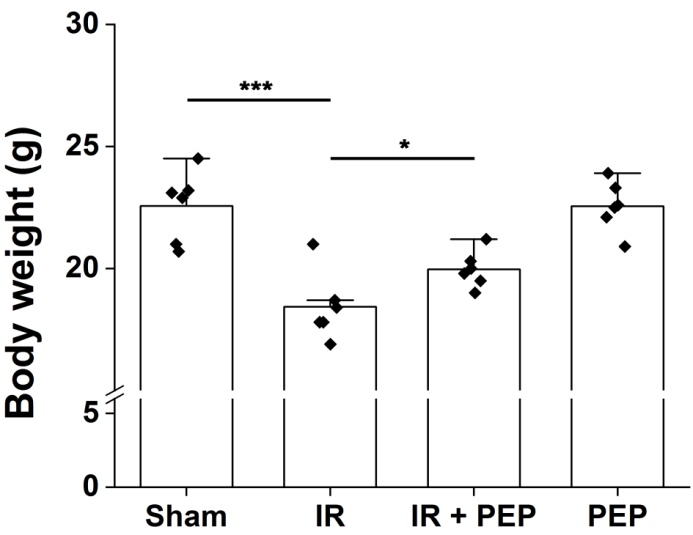


**Figure S2.** Body weights of mice on Day 5 after TAI. Results are shown as means ± SD. ^*^, *P* < 0.05; ^***^. *P* < 0.001.


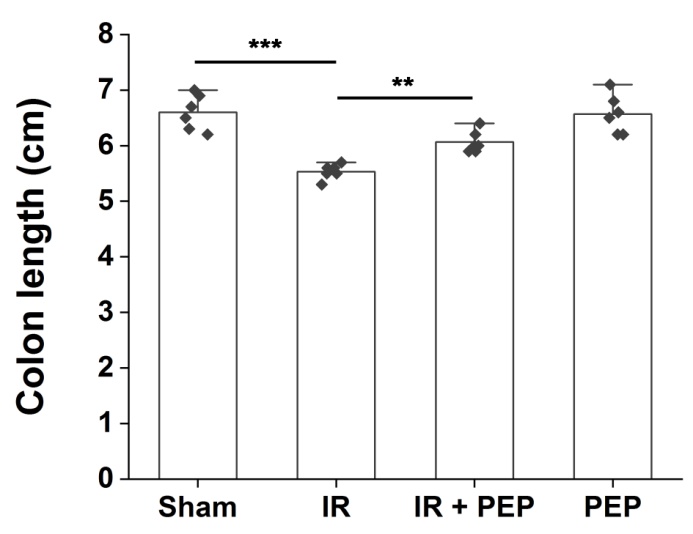


**Figure S3.** Colon length of TAI mice in the absence and presence of ^T7E21R^HD5 treatment. ^**^, *P* < 0.01; ^***^, *P* < 0.001.


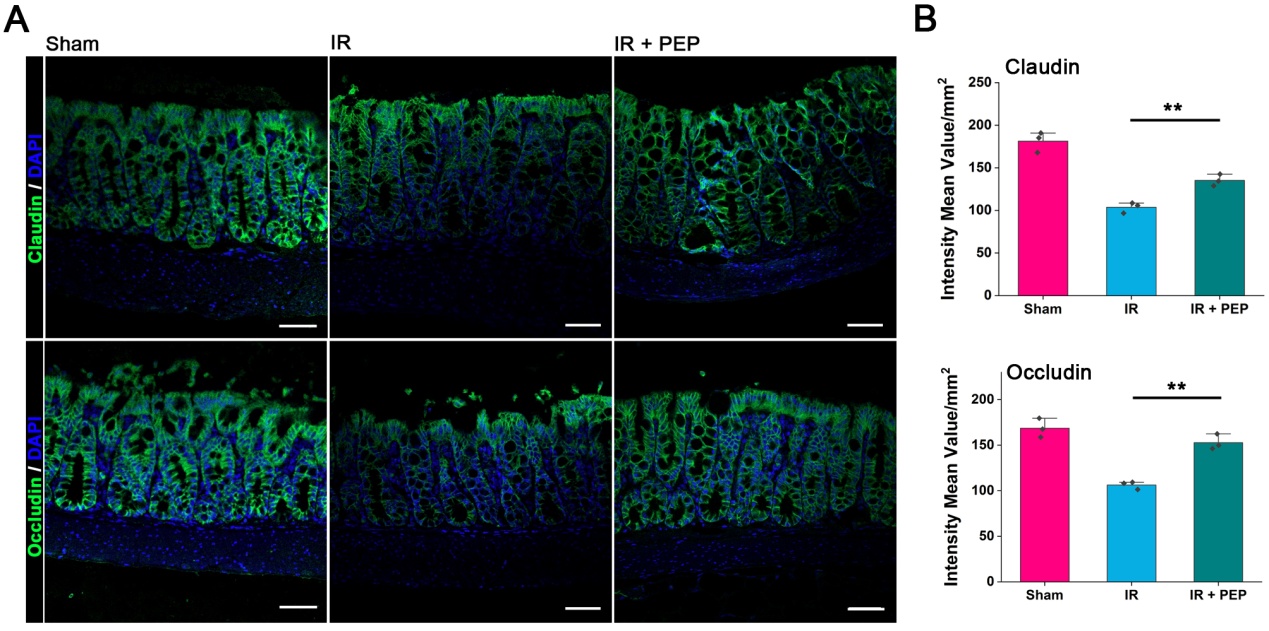


**Figure S4. Amelioration of ^T7E21R^HD5 on the reduction of tight junction proteins in colon induced by IR.** (A) Confocal images indicating the expressions of claudin and occludin in mouse colon. Nucleus is stained by DAPI. The scale bar indicates 20 µm. (B) Mean fluorescence intensity of claudin and occludin. Results are presented as the means ± SD. ^**^, *P* < 0.01.


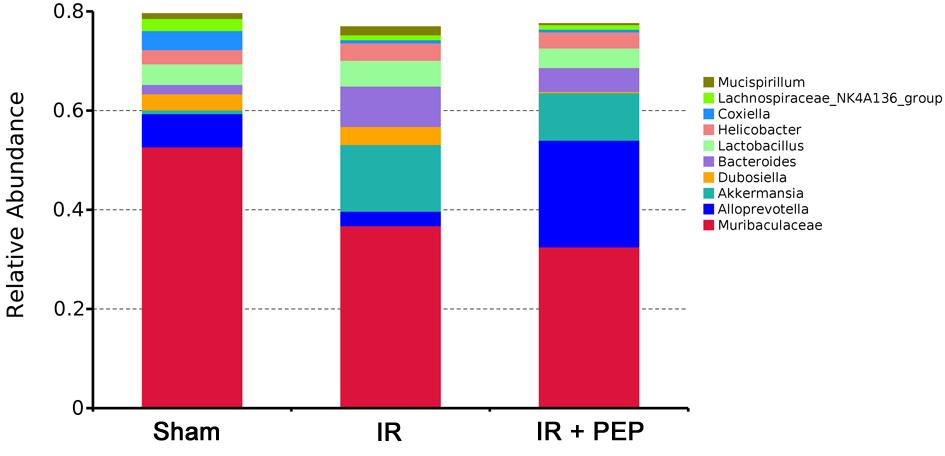


**Figure S5.** Species abundance histogram showing the top 10 bacterial species at the genus level.
